# Heterologous expression of the honeysuckle R2R3-MYB transcription factor LjMYB106 enhances phenylpropanoid and flavonoid metabolism in *Arabidopsis thaliana* and *Nicotiana benthamiana*

**DOI:** 10.3389/fpls.2026.1896352

**Published:** 2026-07-27

**Authors:** Jiangxin Yang, Jingjie Zhang, Xinghua Nie, Huan Shi, Qiaoqiao Xiao, Jiaotong Yang

**Affiliations:** Resource Institute for Chinese and Ethnic Materia Medica, Guizhou University of Traditional Chinese Medicine, Guizhou, China

**Keywords:** flavonoids, LjMYB106, lonicera japonica, transcription factor, transcriptomic and widely targeted metabolomic analyses

## Abstract

*Lonicera japonica* Thunb. is a traditional medicinal plant rich in bioactive flavonoids, but the transcriptional regulation of flavonoid biosynthesis remains unclear. In this study, we identified LjMYB106, an R2R3-MYB transcription factor associated with flavonoid accumulation, from floral developmental transcriptomes. Sequence and phylogenetic analyses showed that LjMYB106 contains conserved R2 and R3 MYB domains and is closely related to homologous MYB proteins. Heterologous overexpression of *LjMYB106* in *Arabidopsis thaliana* and *Nicotiana benthamiana* increased total flavonoid and p-coumaric acid contents. Integrated transcriptomic and widely targeted metabolomic analyses in *A. thaliana* showed that *LjMYB106* overexpression was associated with changes in phenylpropanoid and flavonoid biosynthetic pathways. qRT-PCR validation further showed that *PAL*, *C4H*, *4CL*, *CHS*, *DFR*, and *ANS* homologs were up-regulated, whereas *CHI*, *F3H*, and *FLS* homologs were down-regulated in *LjMYB106*-overexpressing plants. Subcellular localization and yeast transactivation assays indicated that LjMYB106 is a nuclear protein with transcriptional activation activity. These results suggest that LjMYB106 is a candidate regulator of phenylpropanoid and flavonoid metabolism and provide a basis for further studies of MYB-mediated flavonoid regulation in *L. japonica*.

## 1 Introduction

*Lonicera japonica* Thunb., a perennial semi-evergreen vine belonging to the genus *Lonicera* in the Caprifoliaceae family, is a well-known medicinal plant. Its medicinal components are mainly derived from dried flower buds and newly opened flowers of *Lonicera* species (Yang et al., 2023). Pharmacological studies have shown that *L. japonica* possesses multiple biological activities, including antioxidant, anti-inflammatory, antiviral, immunomodulatory, and hepatoprotective effects (Ge et al., 2022). These activities are closely associated with its abundant secondary metabolites. To date, more than 140 secondary metabolites have been identified from *L. japonica*, including phenolic acids, flavonoids, volatile oils, and triterpenoid saponins (Shang et al., 2011; Chai et al., 2005; Chen et al., 2005; Lu et al., 2004). Among these compounds, flavonoids represent an important class of bioactive constituents and are closely related to the medicinal value and quality evaluation of *L. japonica* (Qi et al., 2019). Therefore, elucidating the regulatory mechanisms underlying flavonoid biosynthesis is important for understanding the formation of bioactive constituents in this medicinal plant.

Flavonoids are ubiquitous polyphenolic secondary metabolites in plants. They not only serve as important active constituents in medicinal plants but also play essential roles in plant growth and development, such as flower coloration and pollen fertility, as well as in stress responses, including protection against ultraviolet radiation and pathogen attack (Pourcel et al., 2007). According to structural differences, flavonoids can be classified into several subclasses, including flavanones, flavones, flavonols, proanthocyanidins, anthocyanins, and isoflavones, each with distinct metabolic routes and physiological functions (Winkel-Shirley, 2001; Lepiniec et al., 2006). Flavonoid biosynthesis is initiated from the conserved phenylpropanoid pathway and subsequently proceeds through the catalytic actions of key enzymes, such as chalcone synthase (*CHS*), chalcone isomerase (*CHI*), and flavanone 3-hydroxylase (*F3H*), leading to the formation of diverse flavonoid compounds (Bu et al., 2021). This pathway has been extensively studied in the model plant *A. thaliana*, in which many structural genes involved in flavonoid biosynthesis, including *CHS*, *F3H*, and *DFR*, have been functionally characterized (Saito et al., 2013). Therefore, *A. thaliana* provides a useful system for investigating flavonoid biosynthesis and for evaluating the functions of regulatory genes from medicinal plants.

Transcription factors play central roles in regulating flavonoid biosynthesis. Among them, the MYB-*bHLH*-WDR (MBW) complex, composed of MYB transcription factors, basic helix–loop–helix (*bHLH*) proteins, and *WD40* repeat proteins, has been established as an important regulatory module in flavonoid metabolism (Xu et al., 2015). MYB transcription factors are particularly important because they can activate or repress the expression of structural genes in the flavonoid pathway and thereby influence metabolic flux toward specific flavonoid subclasses, such as flavonols and anthocyanins (Gonzalez et al., 2008).

Functional characterization of MYB homologs in multiple plant species has demonstrated their conserved roles in regulating flavonoid biosynthesis. In *Arabidopsis*, MYB11, MYB12, and MYB111 are classical flavonol regulators. These closely related subgroup 7 R2R3-MYB transcription factors show functional redundancy and activate early flavonoid biosynthetic genes, including *CHS*, *CHI*, *F3H*, and *FLS1*, thereby promoting flavonol accumulation (Stracke et al., 2017, Stracke et al., 2007). By contrast, *PAP1*/MYB75 is mainly involved in the regulation of anthocyanin biosynthesis (Gonzalez et al., 2008; Teng et al., 2005). In *Scutellaria baicalensis*, R2R3-MYB transcription factors bind to promoters of flavone synthase genes to modulate baicalin biosynthesis (Fang et al., 2023). Overexpression of SsMYB106 promoted the accumulation of flavonoids, especially catechins, and upregulated the expression of most structural genes in the flavonoid biosynthetic pathway in *Spatholobus suberectus* (Qin et al., 2025). Together, these studies demonstrate that MYB-related transcription factors are conserved regulators of flavonoid biosynthesis across plant species, with different MYB members contributing to distinct flavonoid branches.

In *L. japonica*, MYB transcription factor studies related to flavonoid biosynthesis remain limited (Liao et al., 2025). Flavonoid accumulation in *L. japonica* changes during flower development (Li et al., 2019), and *MYB106* shows a similar expression pattern; however, the biological function of *LjMYB106* and its role in flavonoid biosynthesis remain unclear. Thus, further functional validation is needed to clarify the MYB-mediated regulatory network underlying flavonoid accumulation in *L. japonica*. Integrated transcriptomic and metabolomic analysis links gene expression changes with metabolite accumulation and is useful for identifying pathways and candidate regulators associated with secondary metabolite biosynthesis (Xue et al., 2022; Zhang et al., 2024; Wang et al., 2021). This strategy has been applied to medicinal plants, including *Artemisia argyi* and *Curcuma wenyujin* (Yi et al., 2021; Chen et al., 2022). In this study, we isolated *LjMYB106* from *L. japonica* and hypothesized that *LjMYB106* is associated with phenylpropanoid and flavonoid metabolism by affecting pathway-related structural genes. We aimed to determine whether heterologous overexpression of *LjMYB106* in *A. thaliana* and *N. benthamiana* enhances total flavonoid and p-coumaric acid accumulation and to identify the genes, metabolites, and pathways associated with *LjMYB106 overexpression* through integrated transcriptomic and metabolomic analyses.

## 2 Materials and methods

### 2.1 Identification of flavonoid-associated MYB candidate genes in *L. japonica*

To identify MYB transcription factors potentially associated with flavonoid accumulation during flower development in *L. japonica*, we retrieved a public transcriptome dataset from the NCBI Sequence Read Archive (accession SRP417164). The dataset included green floral buds, white floral buds, and open flowers, with three biological replicates for each stage. Pairwise comparisons were performed between green floral buds and open flowers and between white floral buds and open flowers. Differentially expressed *MYB* genes were screened using |log2 fold change| > 1 and *P* < 0.05 based on Student’s t-test. *MYB* genes that overlapped between the two comparisons were further examined across the three developmental stages. Genes showing a continuous decrease from green floral buds to white floral buds and open flowers were considered candidate MYB regulators potentially associated with flavonoid accumulation.

### 2.2 Bioinformatic analysis of LjMYB106

The protein sequence of LjMYB106 was used as a query to identify homologous MYB proteins from representative plant species, including *A. thaliana*, *Oryza sativa*, *Sorghum bicolor*, *Setaria italica*, *Glycine max*, and *Brassica napus*, using BLAST searches against the Phytozome database (Goodstein et al., 2012). The two best matched homologous sequences were selected for multiple sequence alignment using the MUSCLE algorithm (Edgar, 2004) implemented in MEGA 12 (Kumar et al., 2024). The homologous MYB protein sequences used for multiple sequence alignment and phylogenetic analysis are listed in **Supplementary Table 1**. Conserved domain analysis was performed using the hmmsearch program in HMMER v3.4 (Eddy, 2011) based on the hidden Markov model of the MYB DNA-binding domain (PF00249) obtained from the InterPro database (Blum et al., 2025).

### 2.3 Cloning of the *LjMYB106* gene and transformation

The coding sequence (CDS) of *LjMYB106* from *L. japonica* was cloned by RT-PCR using the following primers: forward, 5′-ggactctagaggatccccgggATGGGGAGATCTCCATGCT-3′; reverse, 5′-cgatcggggaaattcgagctcTTAGAACACGGGCGAATCAG-3′. PCR was performed with an annealing temperature of 60 °C and yielded a 1,074 bp amplicon. The amplified *LjMYB106* fragment was inserted into the CaMV 35S promoter-driven pBI121 vector to generate the *LjMYB106* overexpression construct. The recombinant vector was introduced into Agrobacterium tumefaciens. Wild-type *A. thaliana* and *N. benthamiana* plants were transformed using the floral dip and leaf-disc transformation methods, respectively. Transgenic plants were screened and verified by PCR and qRT-PCR.

### 2.4 Determination of total flavonoid and p-coumaric acid contents

The contents of total flavonoids and p-coumaric acid were determined in wild-type and *LjMYB106*-overexpressing plants according to previous studies (Karthikeyan et al., 2015; Zhao et al., 2018). p-Coumaric acid content was quantified by high-performance liquid chromatography (HPLC). Approximately 0.1 g of each sample was extracted with 10 mL methanol by ultrasonication for 30 min. The extract was filtered through a 0.22 μm membrane and analyzed using an Agilent HPLC system equipped with an Agilent Polaris 5 C18-A column (250 mm × 4.6 mm, 5 μm). The mobile phase consisted of acetonitrile and phosphoric acid solution (pH 2.4) at a ratio of 20:80 (v/v), with a flow rate of 1.0 mL/min. The column temperature was maintained at 30 °C, the detection wavelength was set at 310 nm, and the injection volume was 10 μL. The p-coumaric acid content was calculated based on a standard curve prepared using authentic p-coumaric acid and expressed as μg/g sample. Total flavonoid content was determined using the aluminium nitrate colorimetric method with rutin as the standard. Briefly, 1.0 g of each sample was extracted with 50 mL of 70% ethanol. An aliquot of the extract was sequentially mixed with NaNO_2_, Al(NO_3_)_3_, and NaOH solutions for color development, and absorbance was measured at 510 nm. Total flavonoid content was calculated from a rutin standard curve and expressed as mg rutin equivalents per gram of sample.

### 2.5 Widely targeted metabolomic analysis

Because *LjMYB106–*2 showed the highest total flavonoid content among the three *A. thaliana* overexpression lines, fifteen-day-old seedlings of WT and the representative line *LjMYB106–*2 were collected for widely targeted metabolomic analysis, with three biological replicates per group. Samples were freeze-dried and ground into powder. For each sample, 25 mg of powder was extracted with 1,000 μL of pre-cooled methanol/acetonitrile/water solution containing internal standards. After vertexing, ultrasonic extraction, low-temperature incubation, and centrifugation, the supernatant was filtered through a 0.22 μm membrane and transferred to LC-MS vials. Widely targeted metabolomic profiling was performed on an LC-MS/MS platform. Metabolites were identified using retention time, ion-pair information, and MS/MS spectra by comparison with an in-house metabolite database and public databases. Metabolites were quantified in multiple reaction monitoring (MRM) mode, and peak areas were integrated and corrected across samples. A pooled quality control (QC) sample was prepared by mixing equal volumes of all extracts and was injected regularly to monitor LC-MS/MS stability and repeatability. Principal component analysis (PCA) and orthogonal partial least squares discriminant analysis (OPLS-DA) were used to evaluate metabolic variation between WT and transgenic samples. Differentially accumulated metabolites (DAMs) were identified using *P* < 0.05 and fold change (FC) ≥ 1.2 or FC ≤ 0.83. KEGG pathway enrichment analysis was then performed to identify significantly enriched metabolic pathways.

### 2.6 RNA extraction, library construction, and transcriptome sequencing

Total RNA was extracted from seedlings of WT and the representative *LjMYB106*-overexpressing line *LjMYB106*-2. RNA quality and integrity were assessed using an Agilent 2100 Bioanalyzer and agarose gel electrophoresis. Qualified RNA samples were used for library construction with the NEBNext^®^ Ultra™ RNA Library Prep Kit (Illumina, USA). Libraries were sequenced on the Illumina NovaSeq 6000 platform by MetWare Biotechnology Co., Ltd. (Wuhan, China). Raw reads were filtered using fastp (Chen et al., 2018) to remove adapters, reads containing more than 10% ambiguous bases, and low-quality reads. Clean reads were used for subsequent transcriptomic analysis. The quality statistics of the transcriptome sequencing data are provided in **Supplementary Table 2**.

### 2.7 Differential gene expression analysis

Clean reads were mapped to the *A. thaliana* TAIR10 reference genome using HISAT2 (Kim et al., 2019). Gene expression levels were quantified using StringTie (Pertea et al., 2015) and normalized as TPM. Gene-level read counts were generated from the StringTie output and used for differential expression analysis with DESeq2 (Love et al., 2014). Genes with |log₂ fold change| ≥ 0.585 and *P* value < 0.05 were defined as differentially expressed genes (*DEGs*). GO and KEGG enrichment analyses of *DEGs* were performed using the clusterProfiler package (Yu et al., 2012) in R. Annotation information from the *A. thaliana* TAIR10 genome was used as the background set. Enriched GO terms and KEGG pathways with an adjusted *P* value < 0.05 were considered statistically significant.

### 2.8 Integrated transcriptomic and metabolomic analysis

Integrated transcriptomic and metabolomic analyses were performed using KEGG enrichment results for differentially expressed genes (DEGs) and differentially accumulated metabolites (DAMs). Shared KEGG pathways enriched in both datasets were identified, and the corresponding genes and metabolites were extracted for joint interpretation of the transcriptional and metabolic changes associated with *LjMYB106* overexpression.

### 2.9 qRT-PCR validation of transcriptomic data

Representative genes from nine flavonoid biosynthesis-related enzyme families, including *PAL*, *C4H*, *4CL*, *CHS*, *CHI*, *F3H*, *FLS*, *DFR*, and *ANS*, were selected for qRT-PCR validation. *A. thaliana Actin7* (AT3G18780) was used as the internal reference gene, and primer sequences are listed in **Supplementary Table 3**. Total RNA was reverse-transcribed into cDNA using the PrimeScript RT Reagent Kit (TaKaRa, Japan). qRT-PCR was performed on a QuantStudio 6 Flex Real-Time PCR System using TB Green Premix Ex Taq II (TaKaRa, Japan). Each 20 μL reaction contained 10 μL of TB Green Premix Ex Taq II, 0.8 μL of each primer, 2 μL of cDNA template, and 6.4 μL of ddH_2_O. The qRT-PCR program was 95 °C for 30 s, followed by 40 cycles of 95 °C for 5 s and 60 °C for 30 s. Melting curve analysis was used to verify amplification specificity. Relative gene expression was calculated using the 2^−ΔΔCt^ method, and statistical significance was assessed with Student’s t-test.

### 2.10 Subcellular localization of LjMYB106 from *L. japonica*

To determine the subcellular localization of LjMYB106, the full-length *LjMYB106* coding sequence without the stop codon was inserted into the pCAMBIA1300-GFP vector to generate the *LjMYB106*-GFP fusion construct. The empty pCAMBIA1300-GFP vector was used as a control. Recombinant plasmids were transformed into *Agrobacterium tumefaciens* strain GV3101 and transiently expressed in *N. benthamiana* leaves. An mCherry-tagged nuclear marker was co-infiltrated to indicate the nucleus. After infiltration, leaves were incubated in darkness under high humidity for 48 h. Fluorescence signals were observed using a laser scanning confocal microscope.

### 2.11 Yeast transactivation activity assay of LjMYB106

The full-length *LjMYB106* coding sequence was inserted into the pGBKT7 bait vector to generate pGBKT7-*LjMYB106*. The pGBKT7-VP16-*LjMYB106* fusion vector was constructed as a positive reference. Empty pGBKT7 served as the negative control, and pGBKT7-VP16 was used as the positive control. All four constructs were transformed into competent Y2HGold yeast cells. Positive transformants were cultured in liquid SD/-Trp medium to OD_600_ = 0.6–0.8 and serially diluted to OD_600_ values of 0.2, 0.02, 0.002, and 0.0002. Equal volumes of yeast suspension were spotted onto SD/-Trp, SD/-His-Trp, and SD/-His-Trp/Ade solid selective media. Plates were incubated at 30 °C in the dark for 3–5 d before colony growth was recorded.

### 2.12 Statistical analysis

All quantitative data are presented as the mean ± standard deviation (SD). Total flavonoid and p-coumaric acid quantification experiments were performed with three independent biological replicates. For qRT-PCR analysis, three biological replicates were used, and each biological replicate included three technical replicates. Relative gene expression was calculated using the 2−ΔΔCt method. Pairwise comparisons between WT and each transgenic line were performed using Student’s t-test, and *P* < 0.05 was considered statistically significant. For widely targeted metabolomic analysis, DAMs were identified using *P* < 0.05 and FC ≥ 1.2 or FC ≤ 0.83, as described in Section 2.5. For transcriptomic analysis, DEGs were identified using DESeq2, and *P* values were adjusted using the Benjamini-Hochberg method to control the false discovery rate, as described in Section 2.7. GO and KEGG enrichment results with adjusted *P* < 0.05 were considered statistically significant.

## 3 Results

### 3.1 Mining MYB family members potentially involved in flavonoid biosynthesis in *L. japonica*

**Figure 1:**
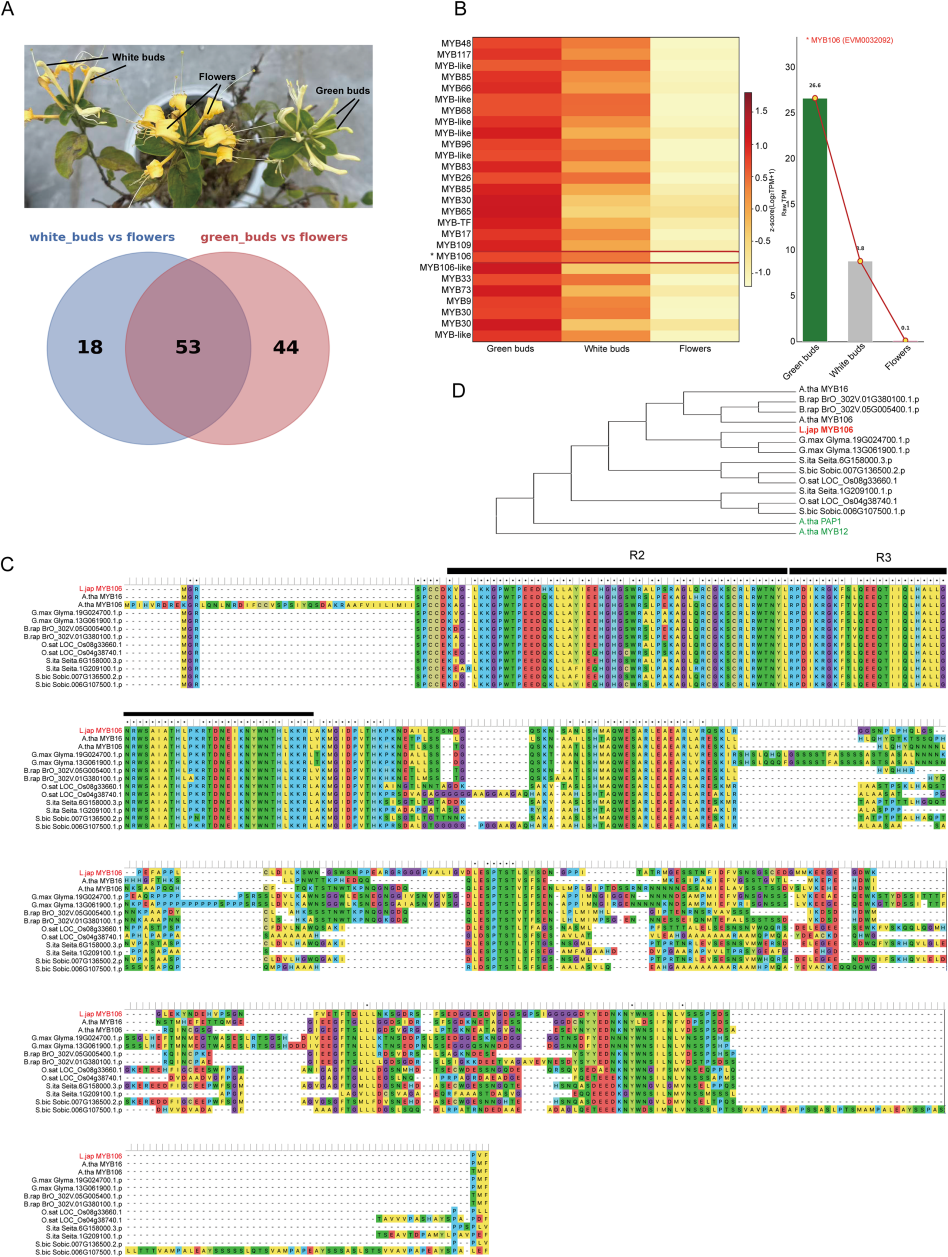
Identification and sequence characterization of LjMYB106 as a candidate flavonoid-related MYB transcription factor in *L. japonica*. **(A)** Venn diagram showing overlapping differentially expressed *MYB* genes from the comparisons of white buds versus flowers and green buds versus flowers. **(B)** Heatmap showing the expression patterns of candidate *MYB* genes across green buds, white buds, and flowers, with LjMYB106 highlighted, green, white, and yellow bars represent green buds, white buds, and yellow buds, respectively. **(C)** Multiple sequence alignment of LjMYB106 and homologous MYB proteins showing the conserved R2 and R3 MYB DNA-binding domains. **(D)** Phylogenetic analysis of LjMYB106 and homologous MYB proteins from representative plant species.

A literature survey showed that flavonoid accumulation in *L. japonica* changes during flower development (Li et al., 2019). Our previous study also identified the *MYB* gene family in *L. japonica* (Zhang et al., 2025). To identify *MYB* genes potentially associated with flavonoid accumulation, we first performed differential expression analysis based on a publicly available transcriptome dataset (NCBI SRA accession: SRP417164), which included three biological replicates each for green floral buds, white floral buds, and open flowers. Differentially expressed *MYB* genes were identified from the comparisons of green floral buds versus open flowers and white floral buds versus open flowers, and the overlapping genes between the two comparisons were retained for further analysis. A total of 53 MYB family members were screened (**Figure 1A**), among which 27 genes exhibited a strictly decreasing expression pattern from green floral buds to white floral buds and then to open flowers (**Figure 1B**; **Supplementary Table 4**).

This expression pattern was consistent with the reported gradual decrease in total flavonoid content during flower development. Notably, *LjMYB106* (EVM0032092) was significantly upregulated in both green floral buds versus open flowers and white floral buds versus open flowers (**Figure 1B**; **Supplementary Figure 1**), showing a bud-preferential expression pattern that was consistent with the spatiotemporal variation in total flavonoid content in *L. japonica*. Considering that SsMYB106, a homologous gene from the closely related medicinal plant *Spatholobus suberectus*, has been reported to positively regulate flavonoid biosynthesis (Qin et al., 2025), we speculated that *LjMYB106* may be a key candidate transcription factor involved in regulating flavonoid accumulation in *L. japonica*.

To characterize the structural features of LjMYB106, its protein sequence was compared with homologous MYB proteins from representative plant species. Multiple sequence alignment showed that LjMYB106 contains two conserved MYB DNA-binding domains corresponding to the R2 and R3 repeats (**Figure 1C**). Phylogenetic analysis placed LjMYB106 with homologs from *Glycine max* and *A. thaliana*, supporting its relationship to R2R3-MYB transcription factors involved in related regulatory processes (**Figure 1D**).

### 3.2 Heterologous overexpression of *LjMYB106* promotes flavonoid and p-coumaric acid accumulation in two model plant species

**Figure 2:**
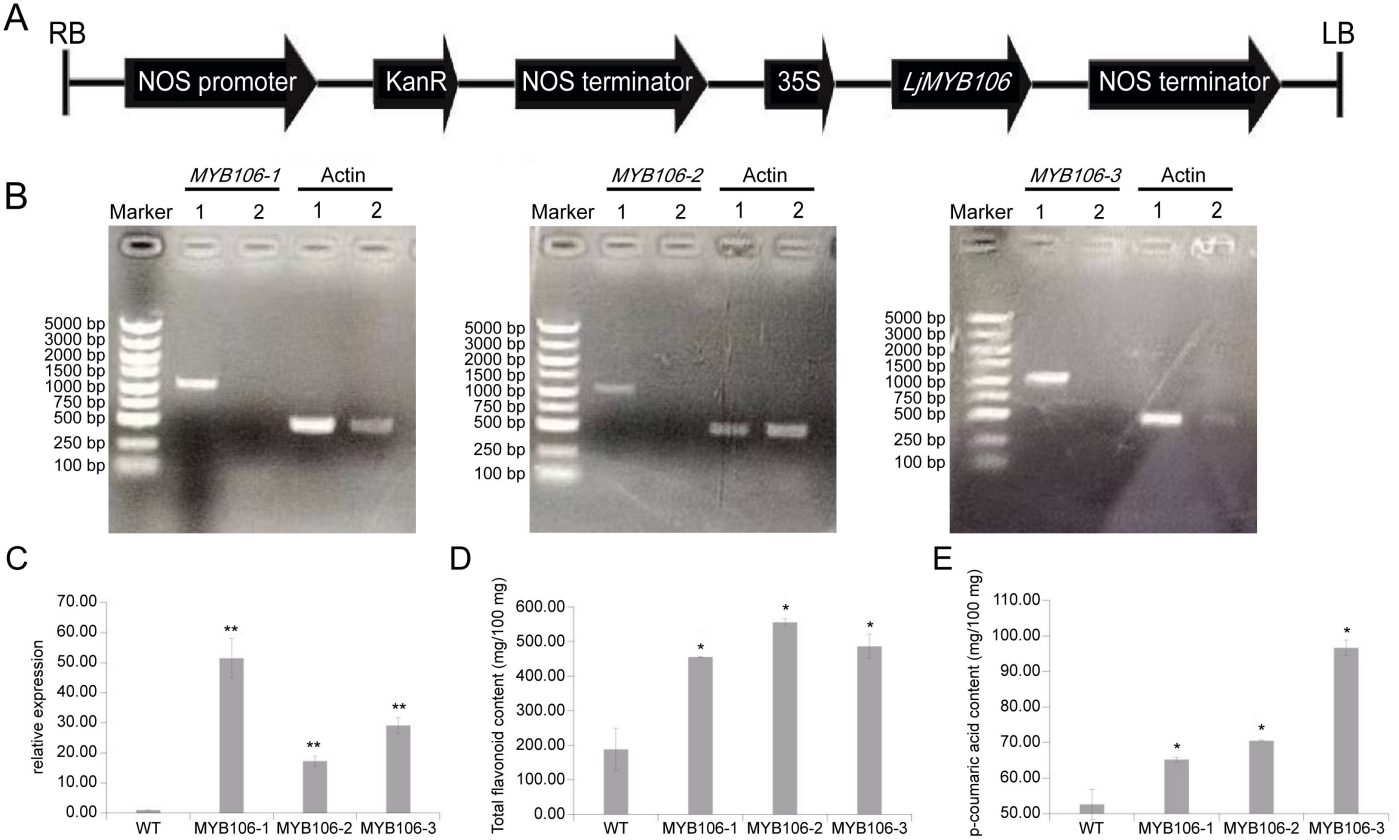
Cloning and functional analysis of LjMYB106 in *A. thaliana*. **(A)** Schematic map of the pBI121-LjMYB106 overexpression vector. **(B)** RT-PCR validation of LjMYB106 expression in transgenic *Arabidopsis* lines by agarose gel electrophoresis. M, 5000 bp DNA marker; MYB106, target gene; Actin, reference gene. **(C)** qRT-PCR analysis of LjMYB106 expression in WT and transgenic lines. **(D)** Total flavonoid content in WT and LjMYB106-overexpressing *Arabidopsis* lines. **(E)** P-coumaric acid content in WT and LjMYB106-overexpressing *Arabidopsis* lines. Error bars indicate SD; n = 3; *P < 0.05; **P < 0.01.

Transgenic *A. thaliana* plants overexpressing *LjMYB106* were screened and validated by PCR and qRT-PCR (**Figures 2A–C**). Compared with WT, *LjMYB106*-overexpressing lines accumulated significantly higher levels of total flavonoids and p-coumaric acid (**Figures 2D, E**). These results indicate that heterologous overexpression of *LjMYB106* promotes total flavonoid and p-coumaric acid accumulation in *A. thaliana*.

Similar phenotypic trends were observed in the heterologous *N. benthamiana* system. RT-PCR and qRT-PCR confirmed *LjMYB106* expression in transgenic lines (**Supplementary Figures 2A, B**). Total flavonoid and p-coumaric acid contents were also markedly higher in *LjMYB106*-overexpressing lines than in WT plants (**Supplementary Figures 2C, D**), suggesting that *LjMYB106* is associated with increased phenylpropanoid and flavonoid metabolism in heterologous plant systems. Representative phenotype images showed the overall growth of WT and *LjMYB106*-overexpressing *N. benthamiana* and *A. thaliana* plants under the experimental conditions (**Supplementary Figures 3A, B**).

### 3.3 *LjMYB106* overexpression remodels the metabolome and affects flavonoid-related metabolites

**Figure 3:**
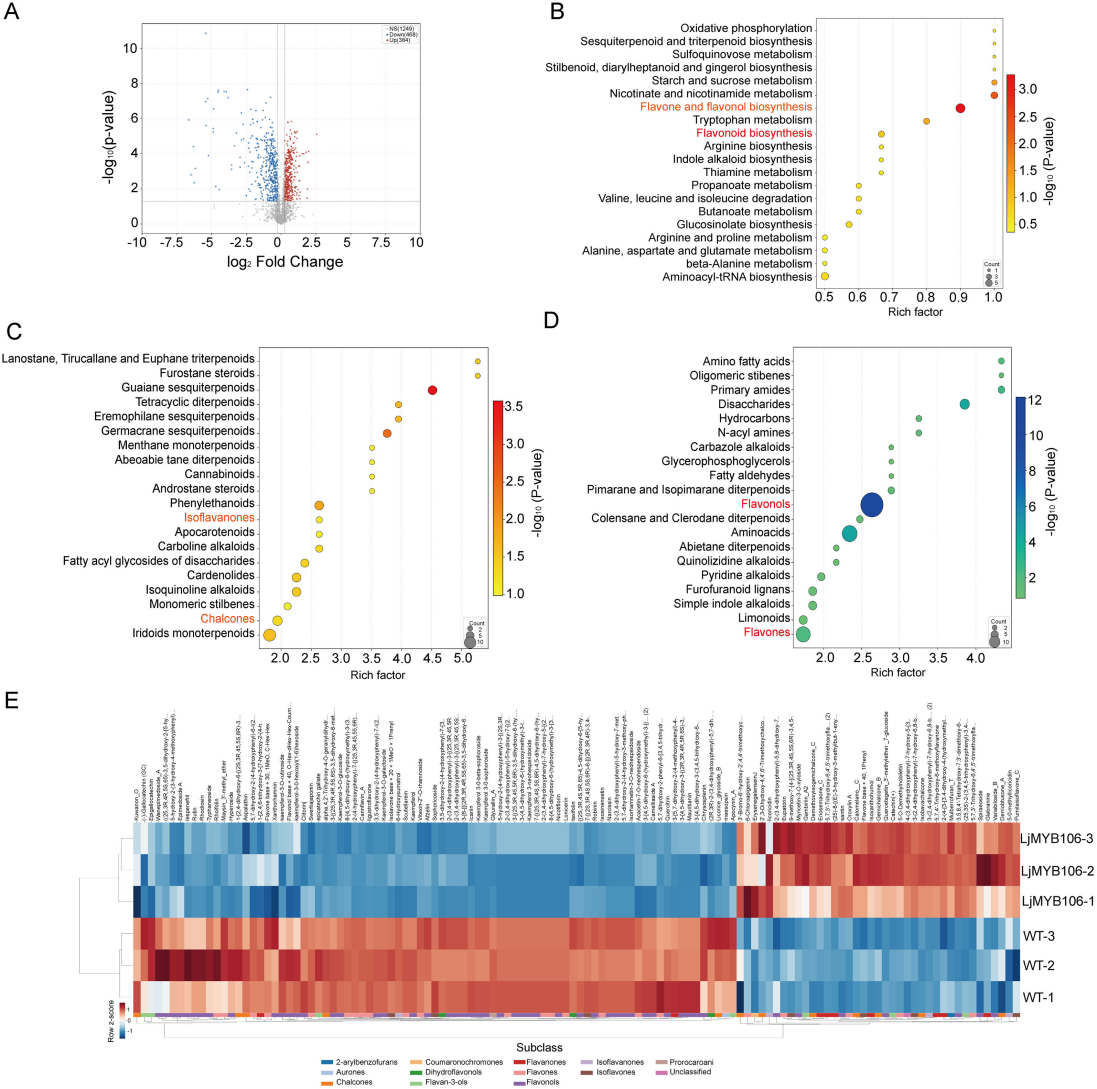
Metabolomic analysis of WT and LjMYB106-overexpressing *Arabidopsis* plants. **(A)** Volcano plot of differentially accumulated metabolites (DAMs) between WT and LjMYB106-overexpressing plants. **(B)** KEGG pathway enrichment analysis of all DAMs. **(C)** Subclass enrichment analysis of up-regulated DAMs. **(D)** Subclass enrichment analysis of down-regulated DAMs. **(E)** Hierarchical clustering heatmap of flavonoid-related DAMs across three LjMYB106-overexpression samples and WT.

Widely targeted metabolomic profiling was performed between WT and *LjMYB106–*2 plants. PCA and OPLS-DA showed clear separation between WT and *LjMYB106*-overexpressing samples (**Supplementary Figure 4**). In total, 2,101 compounds were retained for statistical analysis. 852 differentially accumulated metabolites (DAMs) were identified, including 384 up-regulated and 468 down-regulated compounds in *LjMYB106*-OE relative to WT (**Figure 3A**; **Supplementary Table 5**). KEGG enrichment analysis of all DAMs identified flavone and flavonol biosynthesis and flavonoid biosynthesis as significantly enriched pathways (**Figure 3B**). Among up-regulated DAMs, flavonoid biosynthesis emerged as a candidate pathway but was not significantly enriched in the KEGG-based analysis. This pattern may reflect the absence of KEGG Compound IDs for many differentially accumulated flavonoids, which prevents their inclusion in compound-ID-based enrichment tests. We therefore reclassified all differential metabolites by structural subclass and performed a hypergeometric metabolite set enrichment analysis using subclass as the functional category, with Benjamini-Hochberg FDR correction across all tested subclasses.

Among up-regulated DAMs, flavonoid-related subclasses showed the highest rich factors among the categories tested, including chalcones and isoflavanones, with flavanones showing a similar trend (**Figure 3C**). Among down-regulated DAMs, flavonols were the most significantly over-represented subclass, followed by flavones (**Figure 3D**). This subclass-resolved pattern suggests that *LjMYB106* overexpression is associated with branch-dependent changes in flavonoid metabolism, including increased accumulation of some upstream chalcone- and isoflavanone-type metabolites and reduced accumulation of some flavonol-related metabolites.

Hierarchical clustering was then performed on all flavonoid DAMs to examine subclass-level responses (**Supplementary Table 6**). The heatmap resolved two major expression blocks (**Figure 3E**). The upper cluster was enriched in flavanones, dihydroflavonols, and chalcones that were predominantly down-regulated in *LjMYB106*-2. The lower cluster included flavonols, flavones, and isoflavonoids with mixed regulatory patterns. Several proanthocyanidin-related flavan-3-ol metabolites were elevated in *LjMYB106*-2, consistent with a possible redistribution of flavonoid metabolism among downstream branches.

### 3.4 Transcriptomic analysis links *LjMYB106* overexpression to phenylpropanoid and flavonoid pathway genes

**Figure 4:**
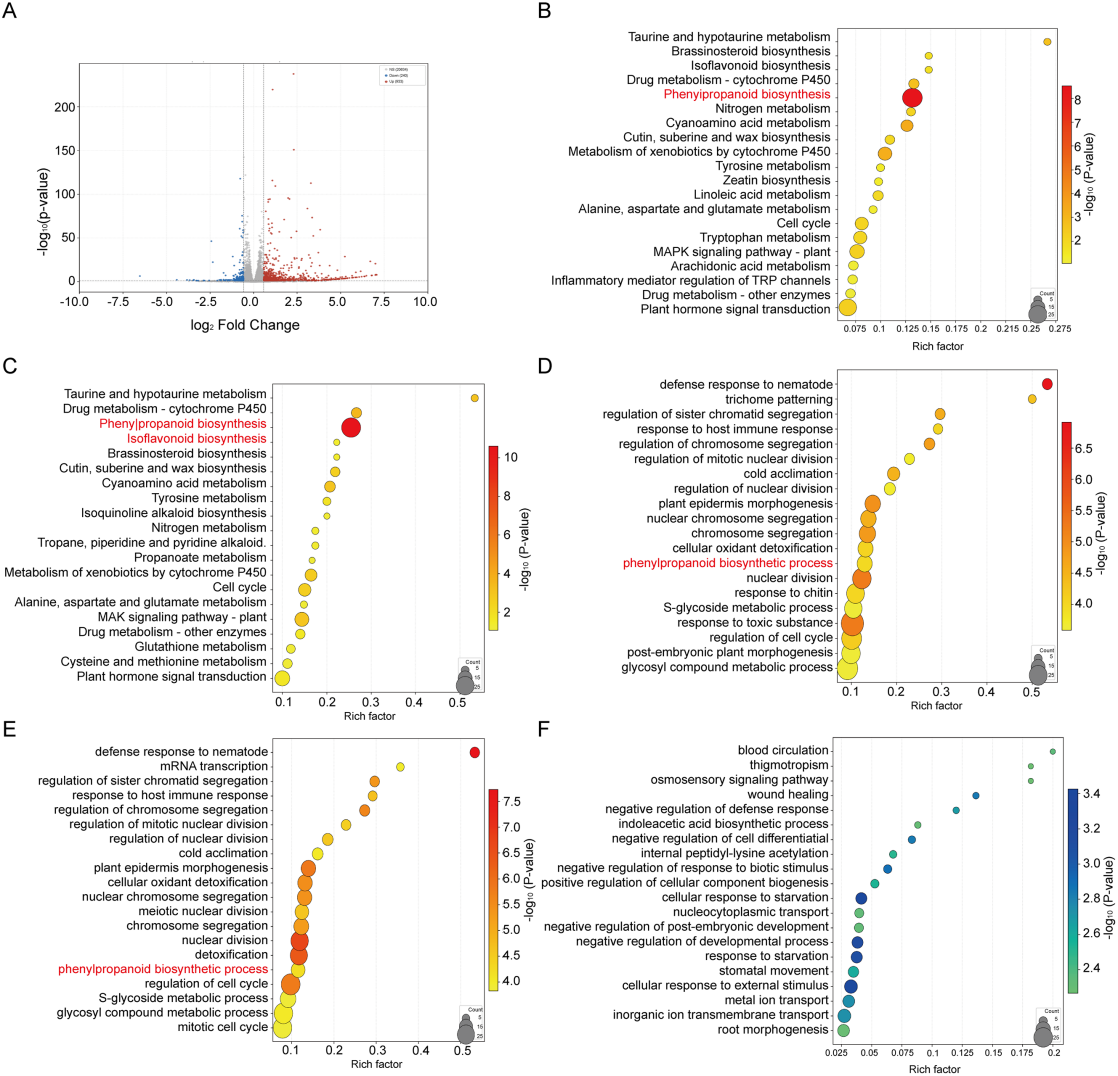
Transcriptomic analysis of WT and LjMYB106-overexpressing *Arabidopsis* plants. **(A)** Volcano plot of differentially expressed genes (DEGs). **(B)** KEGG pathway enrichment analysis of all DEGs. **(C)** KEGG pathway enrichment analysis of up-regulated DEGs. **(D)** GO biological process enrichment analysis of all DEGs. **(E)** GO biological process enrichment analysis of up-regulated DEGs. **(F)** GO biological process enrichment analysis of down-regulated DEGs.

To examine the transcriptional changes associated with *LjMYB106* overexpression, we performed comparative RNA-seq analysis between *LjMYB106*-overexpressing plants and WT. DESeq2 identified 1,173 differentially expressed genes (DEGs), including 933 up-regulated and 240 down-regulated transcripts (**Figure 4A**; **Supplementary Table 7**). KEGG enrichment analysis of all DEGs identified phenylpropanoid biosynthesis as the most significantly enriched pathway, followed by cyanoamino acid metabolism and plant hormone signal transduction (**Figure 4B**). When only up-regulated DEGs were analyzed, phenylpropanoid biosynthesis showed even stronger enrichment, and isoflavonoid biosynthesis emerged as a second significantly enriched flavonoid-related pathway (**Figure 4C**). GO Biological Process enrichment of all DEGs (**Figure 4D**) and up-regulated DEGs (**Figure 4E**) also identified phenylpropanoid biosynthetic process as a significantly enriched term. By contrast, GO terms enriched among down-regulated DEGs (**Figure 4F**) were dominated by abiotic stress response and ion transport, with no phenylpropanoid- or flavonoid-related terms detected.

Together, the KEGG and GO enrichment results indicate that *LjMYB106* overexpression is associated with transcriptional changes in phenylpropanoid and isoflavonoid biosynthetic pathways. These data provide molecular support for a potential role of *LjMYB106* in flavonoid-related gene expression in heterologous *A. thaliana*.

### 3.5 Integrated transcriptomic and metabolomic analysis

**Figure 5:**
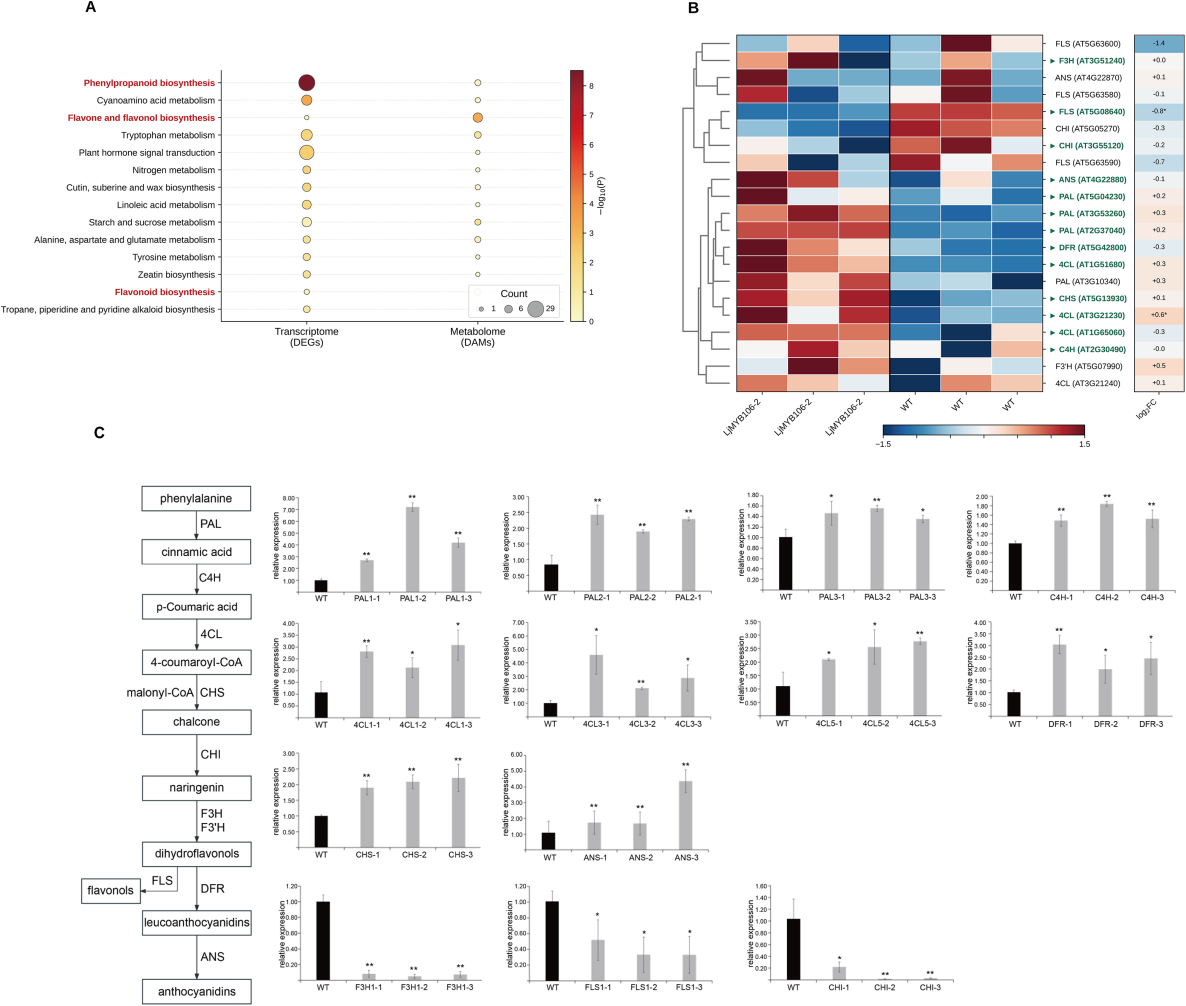
Integrated transcriptomic and metabolomic analysis and qRT-PCR validation of flavonoid biosynthesis-related genes in WT and LjMYB106-overexpressing *Arabidopsis* plants. **(A)** Cross-omics KEGG pathway analysis of differentially expressed genes (DEGs) and differentially accumulated metabolites (DAMs). **(B)** Heatmap showing expression patterns of representative genes involved in phenylpropanoid and flavonoid biosynthesis. **(C)** Schematic representation of the flavonoid biosynthetic pathway and qRT-PCR validation of selected pathway-related genes. *PAL*, phenylalanine ammonia-lyase; *C4H*, cinnamate 4-hydroxylase; *4CL*, 4-coumarate:CoA ligase; *CHS*, chalcone synthase; *CHI*, chalcone isomerase; *F3H*, flavanone 3-hydroxylase; *FLS*, flavonol synthase; *DFR*, dihydroflavonol reductase; *ANS*, anthocyanin synthase. Error bars indicate SD; n = 3; *P < 0.05; **P < 0.01.

To obtain a systems-level view of the metabolic reprogramming associated with *LjMYB106* overexpression, we integrated the differentially expressed genes (DEGs) and differentially accumulated metabolites (DAMs) from the *LjMYB106*-overexpression line *LjMYB106–*2 and WT comparison. Cross-omics KEGG pathway analysis (**Supplementary Table 8**) showed that phenylpropanoid biosynthesis, flavone and flavonol biosynthesis, and flavonoid biosynthesis were commonly represented at both the transcriptomic and metabolomic levels (**Figure 5A**). To place these changes within the flavonoid biosynthetic route, we generated a heatmap of 21 representative paralogous genes encoding key enzymes in the phenylpropanoid and flavonoid pathways (**Figure 5B**). Several upstream phenylpropanoid genes, including PAL, C4H, and 4CL homologs, showed increased expression in *LjMYB106*-overexpressing plants, whereas several downstream flavonol-related genes, particularly FLS homologs, were down-regulated. These expression patterns suggest that *LjMYB106* does not uniformly activate the entire flavonoid pathway but is instead associated with branch-specific modulation of flavonoid biosynthesis. Together, the integrated transcriptomic and metabolomic results indicate that *LjMYB106* overexpression is associated with altered phenylpropanoid or flavonoid metabolic allocation in *A. thaliana*.

### 3.6 qRT-PCR validation of transcriptomic data

To validate the RNA-seq results, representative genes from nine flavonoid biosynthetic enzyme families were selected for qRT-PCR analysis, including *PAL*, *C4H*, *4CL*, *CHS*, *CHI*, *F3H*, *FLS*, *DFR*, and *ANS*. In *LjMYB106*-overexpressing *A. thaliana* lines, most *PAL*, *C4H*, *4CL*, *CHS*, *DFR*, and *ANS* homologs were significantly up-regulated compared with WT, whereas *CHI*, *F3H*, and *FLS* homologs were down-regulated (**Figure 5C**). These trends were generally consistent with the RNA-seq results and supported the reliability of the transcriptomic analysis. The differential regulation of upstream phenylpropanoid genes and downstream flavonoid-branch genes suggests that *LjMYB106* may influence flavonoid metabolism in a branch-dependent manner.

### 3.7 Subcellular localization of LjMYB106

**Figure 6:**
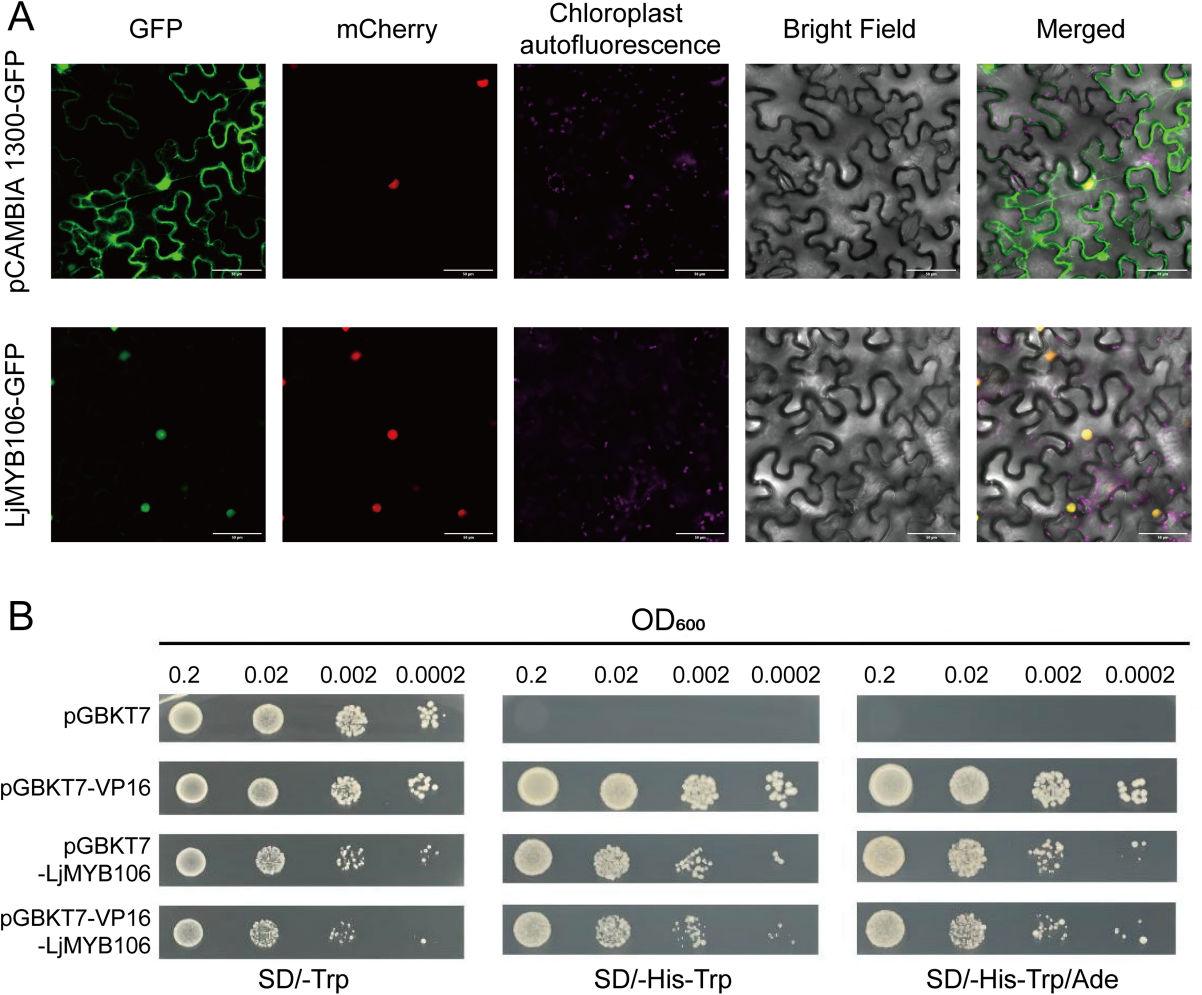
Subcellular localization and transcriptional activation activity of LjMYB106. **(A)** Subcellular localization of LjMYB106-GFP in *N. benthamiana* leaves. GFP, mCherry nuclear marker, chloroplast autofluorescence, bright-field, and merged images are shown. pCAMBIA1300-GFP was used as the control. Scale bars = 50 μm. **(B)** Yeast transcriptional activation assay of LjMYB106 using pGBKT7, pGBKT7-VP16, pGBKT7-LjMYB106, and pGBKT7-VP16-LjMYB106 constructs on SD/-Trp, SD/-His-Trp, and SD/-His-Trp/Ade media at serial OD_600_ dilutions.

To examine the intracellular localization of LjMYB106, the LjMYB106-GFP fusion construct and empty pCAMBIA1300-GFP control were transiently expressed in *N. benthamiana* leaves. GFP from the empty vector was broadly distributed in the cell, whereas LjMYB106-GFP fluorescence was predominantly detected in the nucleus. Co-expression with an mCherry-labelled nuclear marker showed strong overlap between LjMYB106-GFP and the nuclear marker in merged images (**Figure 6A**). These results indicate that LjMYB106 is a nuclear-localized protein, consistent with a potential role as a transcriptional regulator.

### 3.8 LjMYB106 exhibits transcriptional activation activity

All four yeast transformants grew robustly on SD/-Trp medium across the dilution series, confirming successful plasmid transformation. On SD/-His-Trp medium, the negative control pGBKT7 showed little or no growth, whereas the positive control pGBKT7-VP16 grew normally. Yeast carrying pGBKT7-*LjMYB106* showed weak but visible growth, and pGBKT7-VP16-*LjMYB106* showed strong growth comparable to the positive control. On the more stringent SD/-His-Trp/Ade medium, clear colonies were observed for pGBKT7-VP16 and pGBKT7-VP16-*LjMYB106*, whereas pGBKT7 and pGBKT7-LjMYB106 showed little or no growth. These results indicate that LjMYB106 has intrinsic transcriptional activation activity in yeast, and that its transactivation capacity can be enhanced by VP16 fusion (**Figure 6B**).

## 4 Discussion

### 4.1 *LjMYB106* may positively regulate phenylpropanoid and flavonoid accumulation in heterologous systems

Flavonoids are important bioactive metabolites in *L. japonica*, and their biosynthesis is closely associated with the phenylpropanoid pathway (Cao et al., 2023; Liu et al., 2021). In this study, heterologous overexpression of *LjMYB106* in *A. thaliana* and *N. benthamiana* increased the accumulation of total flavonoids and p-coumaric acid, suggesting that *LjMYB106* may play a positive role in phenylpropanoid and flavonoid metabolism. As *p*-coumaric acid is an important intermediate of the phenylpropanoid pathway and serves as a precursor for downstream flavonoid biosynthesis, its increased accumulation in *LjMYB106*-overexpressing plants further supports the potential involvement of *LjMYB106* in this metabolic network.

### 4.2 Integrated transcriptomic and metabolomic analysis reveals *LjMYB106*-mediated reprogramming of the flavonoid metabolic network

Widely targeted metabolomic analysis revealed clear metabolic differences between WT and LjMYB106-overexpressing *A. thaliana* plants. Of 2,101 compounds retained for statistical analysis, 852 were identified as DAMs. KEGG enrichment analysis associated these DAMs with phenylpropanoid and flavonoid-related pathways, including phenylpropanoid biosynthesis, flavonoid biosynthesis, and flavone and flavonol biosynthesis. Transcriptome analysis also showed that *LjMYB106* overexpression altered the expression of genes involved in phenylpropanoid and flavonoid biosynthesis. A total of 1,173 *DEGs* were identified, with most of them being upregulated. Notably, several upstream structural genes, including *PAL*, *C4H*, and *4CL*, were upregulated in *LjMYB106*-overexpressing lines. These genes are involved in the early steps of the phenylpropanoid pathway and provide precursors for downstream flavonoid biosynthesis (Fraser and Chapple, 2011; Vogt, 2010). The increased expression of these upstream genes is consistent with the elevated accumulation of p-coumaric acid and total flavonoids observed in the transgenic plants.

Integrated transcriptomic and metabolomic analyses showed that DEGs and DAMs were co-enriched in pathways related to phenylpropanoid and flavonoid metabolism. This convergence indicates that *LjMYB106* overexpression affects flavonoid-related metabolism at both transcriptional and metabolic levels. In particular, the up-regulation of upstream genes such as *PAL*, *C4H*, and *4CL* was consistent with increased p-coumaric acid accumulation, linking transcriptional changes in early phenylpropanoid metabolism with a corresponding metabolic output.

### 4.3 Comparative analysis with previously characterized MYB regulators

MYB transcription factors are well known regulators of flavonoid biosynthesis in plants (Xu et al., 2015). For example, MYB12 and related R2R3-MYB proteins regulate flavonol biosynthesis (Liao et al., 2025; Mehrtens et al., 2005), whereas *PAP1*/*MYB75* is involved in anthocyanin accumulation (Teng et al., 2005). These studies show that different MYB subgroups may regulate distinct branches of the flavonoid biosynthetic pathway. Although *LjMYB106* is phylogenetically distinct from MYB12 and *PAP1*, its heterologous overexpression in *A. thaliana* and *N. benthamiana* increased the accumulation of total flavonoids and *p*-coumaric acid. Transcriptomic and metabolomic analyses further showed that LjMYB106 overexpression affected phenylpropanoid biosynthesis, flavonoid biosynthesis, and flavone and flavonol biosynthesis pathways. These results suggest that *LjMYB106* may represent a MIXTA-like R2R3-MYB candidate associated with phenylpropanoid/flavonoid metabolic regulation in heterologous systems.

### 4.4 Possible metabolic flux redistribution within the flavonoid biosynthetic pathway

Divergent expression patterns were observed among structural genes involved in phenylpropanoid and flavonoid biosynthesis in *LjMYB106*-overexpressing *A. thaliana*. Several upstream phenylpropanoid-related genes, including *PAL*, *C4H*, and *4CL*, were significantly up-regulated, whereas several midstream or branch-related flavonoid biosynthetic genes, including *CHI*, *F3H*, and *FLS*, were down-regulated. By contrast, the anthocyanin or proanthocyanidin-associated genes *DFR* and *ANS* showed increased transcript levels.

This expression pattern, together with increased total flavonoid content, suggests that *LjMYB106* may not uniformly activate the entire flavonoid biosynthetic pathway. Instead, *LjMYB106* overexpression may enhance the upstream supply of phenylpropanoid precursors while differentially affecting downstream flavonoid branches. Up-regulation of *PAL*, *C4H*, and *4CL* may increase carbon input into the phenylpropanoid pathway, whereas down-regulation of *FLS* may reduce allocation of intermediates toward the flavonol branch. Increased *DFR* and *ANS* expression may also indicate changes in late flavonoid branch gene expression.

Thus, the elevated total flavonoid content in *LjMYB106*-overexpressing plants may be associated with coordinated but branch-dependent changes in the phenylpropanoid or flavonoid network. However, targeted quantification of anthocyanins and proanthocyanidins was not performed, so metabolic flux redistribution remains an interpretation rather than direct evidence. Further targeted metabolite profiling and promoter-binding assays will be needed to determine how *LjMYB106* affects specific branches of flavonoid biosynthesis.

### 4.5 Limitations and future perspectives

Although *LjMYB106* was preliminarily associated with phenylpropanoid and flavonoid metabolism through heterologous overexpression in *A. thaliana* and *N. benthamiana*, the present study has several limitations. All functional assays were performed in heterologous model plants, and no gain- or loss-of-function experiments were conducted in the native host, *L. japonica*. Endogenous overexpression, virus-induced gene silencing (VIGS), RNA interference (RNAi), and CRISPR/Cas9-mediated knockout analyses are therefore needed to clarify the *in vivo* function of LjMYB106 in honeysuckle. Accordingly, the native regulatory role of *LjMYB106* in honeysuckle flavonoid biosynthesis remains unresolved.

Another limitation is that the transcriptomic and metabolomic analyses were performed using only one representative *LjMYB106*-overexpressing line. Although this line showed clear *LjMYB106* overexpression and was selected for omics profiling, the use of a single line may not fully capture line-to-line variation caused by transgene insertion position, expression level, or other background effects. Nevertheless, several key findings derived from the omics analysis were further supported by targeted validation across three independent transgenic lines, including the accumulation of p-coumaric acid and total flavonoids, as well as the expression patterns of selected phenylpropanoid and flavonoid biosynthetic genes detected by qRT-PCR.

In addition, the effects of *LjMYB106* on flavonoid biosynthetic structural genes, including *PAL* and *CHS*, were inferred from multi-omics correlations and heterologous phenotypes. The present study does not provide direct evidence that *LjMYB106* binds to or transcriptionally activates these candidate target genes. Promoter-focused assays, such as EMSA, ChIP-qPCR, yeast one-hybrid assays, and dual-luciferase reporter assays, will be required to distinguish correlative expression changes from direct transcriptional regulation. These experiments will be prioritized in future work to define the molecular mechanism by which *LjMYB106* may regulate downstream structural genes.

Future studies should establish or optimize stable transformation and transient expression systems for *L. japonica* to enable in-planta functional assays. Combining spatiotemporal expression profiling, targeted metabolite analysis, and biochemical interaction assays will help clarify the biological role of *LjMYB106* in regulating flavonoid accumulation in native honeysuckle.

## 5 Conclusion

In this study, *LjMYB106* was identified as a candidate R2R3-MYB transcription factor associated with flavonoid accumulation in *L. japonica* through developmental transcriptome mining, sequence characterization, and phylogenetic analysis. Heterologous overexpression of *LjMYB106* in *A. thaliana* and *N. benthamiana* increased total flavonoid and p-coumaric acid accumulation, indicating that *LjMYB106* is associated with enhanced phenylpropanoid and flavonoid metabolism in heterologous plant systems. Integrated transcriptomic and widely targeted metabolomic analyses in *A. thaliana* further showed that *LjMYB106* overexpression was accompanied by coordinated changes in phenylpropanoid- and flavonoid-related pathways, including altered expression of multiple structural genes and branch-dependent changes in flavonoid-related metabolites. Subcellular localization and yeast transactivation assays supported the characterization of LjMYB106 as a nuclear R2R3-MYB protein with transcriptional activation activity. Together, these findings identify LjMYB106 as a promising candidate regulator of phenylpropanoid/flavonoid metabolism and provide a foundation for future studies of MYB-mediated flavonoid biosynthesis in *L. japonica*. Direct promoter-binding assays and functional validation in native honeysuckle will be required to define the precise regulatory mechanism and biological role of *LjMYB106*.

## Data Availability

The raw RNA-seq data generated in this study have been deposited in the NCBI BioProject database under accession number PRJNA1475172 (https://www.ncbi.nlm.nih.gov/bioproject/PRJNA1475172). Metabolite quantification data, qRT-PCR validation data, and related analytical results are provided in the article and supplementary information files. The raw metabolomics data supporting the findings of this study have been deposited in Figshare under the DOI: 10.6084/m9.figshare.32922983. The dataset contains raw LC-MS data in mzXML format for wild-type and LjMYB106-overexpressing A. thaliana plants, including both positive and negative ion modes, with three biological replicates per group.

## Supplementary material

Supplementary Figure 1 Expression levels of LjMYB106 in different tissues and floral developmental stages of *L. japonica*. Transcript abundance is shown as TPM (transcripts per million).

Supplementary Figure 2 LjMYB106 overexpression increases total flavonoid and p-coumaric acid contents in *N. benthamiana*. **(A)** RT-PCR identification of LjMYB106 expression by agarose gel electrophoresis. M, 5000 bp DNA marker; 1, WT; 2–4, resistant transgenic lines. **(B)** qRT-PCR analysis of LjMYB106 expression in WT and transgenic lines. **(C)** Total flavonoid content in WT and LjMYB106-overexpressing *N. benthamiana* lines. **(D)** P-coumaric acid content in WT and LjMYB106-overexpressing *N. benthamiana* lines. Error bars indicate SD; n = 3; **P* < 0.05; ***P* < 0.01.

Supplementary Figure 3 Representative phenotypes of LjMYB106-overexpressing *N. benthamiana* and *A. thaliana* plants. **(A)** Phenotypes of WT and LjMYB106-overexpressing *N. benthamiana* lines. **(B)** Phenotypes of WT and LjMYB106-overexpressing *A. thaliana* lines.

Supplementary Figure 4 PCA and OPLS-DA analyses of the metabolomes of WT and LjMYB106-overexpressing *Arabidopsis* lines. Left, PCA score plot showing separation between WT and LjMYB106-overexpressing samples. Right, OPLS-DA permutation test plot. Orange circles indicate WT samples, and blue squares indicate LjMYB106-overexpressing lines. R2Y, model goodness-of-fit; Q2, model predictability.
